# Tuberculosis in children perinatally exposed to HIV in the current epidemiological TB context

**DOI:** 10.1186/1471-2334-14-S7-O30

**Published:** 2014-10-15

**Authors:** Delia Vlad, Mariana Mărdărescu, Mihaela Petrea, Sorin Petrea, Florin Murgoci, Cristina Petre, Ana Maria Tudor, Ruxandra Neagu-Drăghicenoiu, Rodica Ungurianu, Alina Cibea, Dan Oțelea, Tatiana Colțan

**Affiliations:** 1 National Institute for Infectious Diseases "Prof. Dr. Matei Balş", Bucharest, Romania; 2 Institute of Pneumology "Marius Nasta", Bucharest, Romania; 3 Carol Davila University of Medicine and Pharmacy, Bucharest, Romania

## Background

Romania is among the top European countries from the European Union in terms of tuberculosis (TB) incidence. Children with perinatal HIV infection represent a special category due to the increasing and more frequent cases of pulmonary and extrapulmonary TB, delayed detection, disease severity, and last but not least the emergence of multi-drug-resistant disease.

## Method

During 01.01.2011 -01.07.2014 in the Immunocompromised Children Department from the National Institute for Infectious Diseases "Prof. Dr. Matei Balş" we analyzed the dynamical evolution of 214 perinatally HIV exposed children, aged 0-4 years. Lack of vaccination due to prematurity and the degree of immunosuppression in maternity contributed considerably to the increased incidence of TB in the family’s epidemiological context (at least one family member is diagnosed with tuberculosis). A troubling aspect is that these children come from families with resistant forms of tuberculosis (TB - MDR) due to their parents’ poor adherence to treatment, mainly those pertaining to the 1989-1993 HIV cohort in Romania.

## Results

Of the total number of exposed children 9.81% were diagnosed with tuberculosis of which 14% in apparent primary TB, 71.4% primary TB, 9.52% secondary TB (i.e. 4.76% with abdominal determination, 4.76% marrow). All children were diagnosed positive following the TB family epidemiological investigation. Of the 214 assessed cases, only 3.27% represented contacted TB who received prophylactic tuberculosis.

## Conclusion

The increased incidence of tuberculosis in the general population, particularly in children from mothers with HIV lead to significant percentages and treatment-resistant forms of the disease. Furthermore this makes it difficult for TB to be eradicated which renders it an important public health issue in Romania.

